# The New Syndicate

**Published:** 1888-07

**Authors:** W. X. Sudduth


### THE NEW SYNDICATE.

In assuming the editorship and business management of the organ of the International Dental Journal Association we feel that we have taken a heavy load of responsibility upon ourselves. We have entered upon the work in the belief that by so doing we can best serve our chosen profession, and with the full knowledge that it is to be largely a labor of love. The grand strides made in dentistry during the past few years seem to demand a journal that shall represent the spirit of progress. No personal interests shall be allowed to interfere with its pronounced mission of working for the best good of the dental profession, which has outgrown the trade journals of the day. They have served a very good purpose in the past, and will continue to supply the demand for which they are intended, viz., the distribution of monthly catalogues.

The success of the present movement is the strongest evidence of the needs and wishes of the profession at the present time. It is no ephemeral growth, but an impression that has steadily increased until it has culminated in this pronounced expression in the interest of independent journalism. The JOURNAL is to be published by the International Dental Journal Association, the stock of which is now on the market. Every man who can possibly afford it, and who has the interest of his profession at heart, should take some stock.

In order to insure that the control of the JOURNAL shall remain in the hands of the profession, none but dentists in actual practice will be allowed to subscribe for stock. The stock is non-transferable, except to the association issuing, and to which it must be sold at its appraised valuation when the party holding it desires to dispose of it, either from pleasure, failure or disease. The association may replace stock so coming into their possession. In order to prevent the control of the JOURNAL getting into the hands of the few, the number of shares that any one person may hold is limited to four.

The management of the JOURNAL is to be in our hands, subject to the supervision of an Executive Committee. The executive officers and committee are to be elected annually. A permanent organization has been formed, and every safeguard has been employed to insure perpetuity. The executive officers for the ensuing year are Louis Jack, President; Benjamin Lord, V. President; Geo. S. Allan, Secretary; C. N. Pierce, Treasurer, and O. E. Hill, Chairman of the Executive Committee. In inaugurating this enterprise we consider ourselves fortunate in having secured a coalescence with THE INDEPENDENT PRACTITIONER. It was only after repeated conferences that Dr. Barrett and the Executive Committee of the N. Y. Journal Association were convinced that our plans gave promise of a wider field of usefulness, and that they could better serve the profession at large by uniting with us. THE INDEPENDENT PRACTIONER has always taken high ground for the good of dentistry, and its columns have been open to contributions that looked toward the elevation of the standard of dental education. Drs. Barrett, Carr, Francis, Hill and Bödecker, who have done most to advance the interests of THE INDEPENDENT PRACTITIONER, have come in heartily with the new association, and while, by the new arrangement, they will be relieved of the arduous duties that formerly devolved upon them, will yet be found actively engaged in promoting the cause of independent journalism. The amount of labor they have given toward establishing the PRACTITIONER upon a solid basis will never be known to any except themselves. The profession owes them a debt of gratitude for their unselfish efforts expended in its behalf. It is to be hoped that the movement will receive more encouragement in the future than in the past. We shall at least do our part in presenting the matter fully before the profession. In order to accomplish this, Dr. Barrett has kindly consented to issue the August and September numbers of the JOURNAL.

The delay in issuing the present number was due to the necessary changes connected with the transfer of the journal to the new syndicate. Hereafter we shall try to mail regularly on the last day of the month. Starting with the INDEPENDENT PRACTITIONER as a basis, it is not our intention to make any radical changes either in the form or policy of the journal until the close of the present volume, when a new name will be chosen, and a marked change in dress will be given to the journal. Enlargements will be made from time to time to meet requirements. We intend to publish a live journal, and one that shall be "in touch" with the profession. After thoroughly discussing the matter it was decided that the best way to do this was to make the journal the absolute property of the profession. The success of the PRACTITIONER has been undoubtedly due to this policy, and we hope, by increasing the number of stockholders, and at the same time adding to the capital stock, to increase its sphere of usefulness. Knowing full well that large bodies are apt to become unwieldly, we have taken every precaution to prevent this by thorough organization. Working committees have been appointed with specified duties to perform. Efforts have been heretofore made to organize the profession, but without marked success. Such movements have been carried on successfully in medical journals, and we see no good reason why the same cannot apply to dental journalism. The success of the PRACTITIONER in the past gives every promise for the future, and we look forward to the day when the PRACTITIONER will be *the* journal in circulation as it is now in merit. To this end we desire to interest as many persons in the movement as possible, and shall endeavor to unite the entire profession in it. We shall make it International in character by appointing correspondents in all foreign cities where men of note can be found. Those who have been most active in promoting the interest of the enterprise are confident that the times are now ripe for the establishment of a first-class journal that shall be untrammelled in its endeavor to advance the cause of higher education. The character and number of the men who have already taken stock in the association give assurance of success from the beginning. One of the surest signs of the growth of the dental profession is this very demand for independent journalism, and in our effort to supply the demand we are but voicing the needs of the profession. That our efforts may be successful we ask your hearty coöperation and considerate indulgence when needed. W. X. SUDDUTH.

